# Evaluation of Optical and Radar Based Motion Capturing Technologies for Characterizing Hand Movement in Rheumatoid Arthritis—A Pilot Study

**DOI:** 10.3390/s21041208

**Published:** 2021-02-09

**Authors:** Uday Phutane, Anna-Maria Liphardt, Johanna Bräunig, Johann Penner, Michael Klebl, Koray Tascilar, Martin Vossiek, Arnd Kleyer, Georg Schett, Sigrid Leyendecker

**Affiliations:** 1 Chair of Applied Dynamics, Friedrich-Alexander-Universität Erlangen-Nürnberg (FAU), 91058 Erlangen, Germany; uday.phutane@fau.de (U.P.); johann.penner@fau.de (J.P.); michael.klebl@fau.de (M.K.); 2 Department of Internal Medicine 3—Rheumatology and Immunology, University Hospital Erlangen, FAU, 91054 Erlangen, Germany; anna-maria.liphardt@uk-erlangen.de (A.-M.L.); koray.tascilar@uk-erlangen.de (K.T.); arnd.kleyer@uk-erlangen.de (A.K.); georg.schett@uk-erlangen.de (G.S.); 3 Institute of Microwaves and Photonics, FAU, 91058 Erlangen, Germany; johanna.braeunig@fau.de (J.B.); martin.vossiek@fau.de (M.V.)

**Keywords:** arthritis, hand movement, hand function, optical measurement system, markerless motion capture, Doppler radar, Moberg-Picking-Up Test

## Abstract

In light of the state-of-the-art treatment options for patients with rheumatoid arthritis (RA), a detailed and early quantification and detection of impaired hand function is desirable to allow personalized treatment regiments and amend currently used subjective patient reported outcome measures. This is the motivation to apply and adapt modern measurement technologies to quantify, assess and analyze human hand movement using a marker-based optoelectronic measurement system (OMS), which has been widely used to measure human motion. We complement these recordings with data from markerless (Doppler radar) sensors and data from both sensor technologies are integrated with clinical outcomes of hand function. The technologies are leveraged to identify hand movement characteristics in RA affected patients in comparison to healthy control subjects, while performing functional tests, such as the Moberg-Picking-Up Test. The results presented discuss the experimental framework and present the limiting factors imposed by the use of marker-based measurements on hand function. The comparison of simple finger motion data, collected by the OMS, to data recorded by a simple continuous wave radar suggests that radar is a promising option for the objective assessment of hand function. Overall, the broad scope of integrating two measurement technologies with traditional clinical tests shows promising potential for developing new pathways in understanding of the role of functional outcomes for the RA pathology.

## 1. Introduction

Rheumatoid arthritis (RA) is a chronic inflammatory auto-immune disease [1]. Patients suffering from RA show arthritis mainly of the finger and hand joints that may result in permanent destruction of the affected skeletal sites. Therapeutic measures aim to control inflammation and avoid irreversible tissue destruction of the affected joints. The diagnosis of RA, and in the course of the disease the assessment of disease activity, is based on biochemical and physical characteristics, which are quantified using serology, physical examination and imaging procedures [2]. Hand function in these patients is impaired because of pain, swelling and joint stiffness. Depending on the level of disease activity, RA patients have lower skeletal muscle function [3]. In the clinical context, functional impairment is mainly assessed using patient reported outcome measures, such as the Health Assessment Questionnaire (HAQ, [4]) for arthritis related physical impairment, visual analog scale (VAS) for global disease activity or hand specific questionnaires, such as the Michigan Hand Questionnaire (MHQ) [5]. Furthermore, simple validated functional tests like isometric grip strength [6] or the Moberg-Picking-Up Test (MPUT, [7]) can be used to quantify muscle performance and fine motor skills but do not allow identifying and quantifying differences in movement patterns. Additionally, these functional measures even though they are objective, often are not well related with the above mentioned subjective patient reported outcome measures and do not provide a resolution for discrepancies between clinical scores and subjective patient reported disease activity. Age and sex both affect subjective and objective clinical hand function outcomes in RA patients as we have shown in a recent study [8]. In light of the state-of-the-art treatment options for RA patients, a detailed and early quantification and detection of disease activity is desirable, see [9], to allow personalized treatment regiments and amend currently used subjective patient reported outcome measures. Hand function can potentially serve as an early indicator of change in disease activity and thus allow timely adaptation of patient management procedures.

The current gold standard for capturing human movements are optoelectronic measurement systems (OMS), see [10]. An OMS sends out light, which is being reflected by optical markers, detects the reflection and estimates the 3D position of the marker using time-of-flight-triangulation. While human gait has been measured and modeled in detail for many years, in the reference of RA [11,12,13], comprehensive biomechanical description of hand movement is sparse, with a few limited examples [14,15]. The assessment of hand movement is complex due to the high number of degrees of freedom resulting from the various finger and hand joints [16]. Thus, previous studies mainly investigated individual fingers or very specific movements [17,18,19]. A detailed quantification of hand function capturing simple tasks but also complex movements that can reflect subjectively observed hand function impairment in patients with RA would be desirable. While achieving a detailed kinematic description of hand function in RA patients based on OMS data is the first step for a biomechanical characterization of hand function, data collection with these methods is time consuming and requires a well equipped laboratory with optimal light conditions. Furthermore, hand movement may be artificially changed because of restrictions due to markers mounted on the skin and the artificial test environment. To acquire hand function in a more natural environment, markerless capturing of movement is desirable.

In this context, Doppler radar is an attractive option for recording movement without markers and independent of lighting conditions [20]. A Doppler radar sends out an electromagnetic wave and measures its reflection. The received signal thus contains the Doppler frequency shift $f_{D}$, which is caused by a target moving towards or away from the radar at specific speed. It can be calculated by $f_{D}\approx 2v/\lambda$, where λ is the wavelength of the carrier signal and *v* is the speed radial to the line of sight of the radar. Further modulations, which are called micro-Doppler signatures, arise in the presence of additional micro-motions and overlay the Doppler signal [21]. Doppler radars are capable of measuring the speed of a target *v* by evaluating the Doppler frequency shift $f_{D}$ and in the last decades have been used to detect various motion patterns, e.g., vital signs such as heart and respiratory rate [22,23,24,25,26]. Furthermore, Range-Doppler-Maps have been used to detect falls [27,28]. Besides that, Doppler radar can be used for gait analysis [29,30,31,32]. In addition, Doppler radar and the micro-Doppler envelopes measured with it are also used for hand gesture recognition [33,34,35,36]. The previously mentioned examples of Doppler radar usage to measure different human motions differ regarding the used radar waveform. A continuous wave (CW) radar without any frequency modulation continuously sends out an electromagnetic wave at a constant radio frequency, which does not allow any spatial resolution. Frequency-modulated CW radars are capable to detect the time of arrival of the different wave parts and thus allow spatial resolution in the range dimension (parallel to the line of sight). The same also applies to pulsed waveforms [37]. Radar systems also differ in the number of transmit and receive antennas used. Monostatic radars only have one antenna that serves as transmit and receive antenna. Bistatic radars have one antenna for sending and receiving [38]. Furthermore, many modern radar systems use antenna arrays to transmit and receive the radar signals. They are called multistatic or MIMO (multiple input multiple output) radars and allow angular resolution (orthogonal to the line of sight) [39]. A review of how these different radar waveforms and architectures can be used for sensing human life activities can be found in [40]. In this study, a simple monostatic 24 GHz CW Doppler radar, which was originally designed as a vital signal radar, was used to examine finger motion. The received signal contains a superposition of all reflections of the object scene. Because of that, the main challenge when measuring hand motion using a monostatic CW radar compared to using an OMS is to detect which signal components are caused by which hand or body part.

In addition to measuring kinematics, the use of electromyography (EMG) measurement technologies to understand effects of arthritis has been extensive in human gait motion, e.g., Refs. [41,42] to name a few. Therefore, it is desirable to quantify the grip strength across RA patients and healthy subjects in terms of muscle activity to understand possible effects of RA on the musculotendon structure of the hand as done for a few studies, see [43,44].

The overall challenge for this integrative experimental approach is to identify hand movements that are ascertainable with all here applied methods, the clinical parameters, OMS and radar technology and at the same time are relevant for characterizing hand movement in RA patients. Thus, the objectives of this study were to (1) evaluate the potential of radar as a markerless technology for capturing human motion, (2) use OMS to identify relevant hand movements that better differentiate RA patients from healthy controls compared to clinical tests (grip strength and MPUT times) and characterize impairment more detailed than the questionnaires (e.g., HAQ), (3) design a setup that allows to use OMS and radar in parallel and is suitable for data acquisition with patients from a very early stage during development of the technology, (4) to evaluate the potential of future radar developments to replace OMS in the investigation of hand movement in the long run. We are clearly at the very beginning of designing an experimental setup with the goal to further develop the suggested technologies.

## 2. Methods

### 2.1. Experimental Setup

#### 2.1.1. Subject Characteristics and Clinical Hand Function Assessment

Individuals diagnosed with RA (ACR/EULAR 2010 criteria [2]) and healthy controls were included in the study. Patient participants were recruited from the Internal Medicine 3—Rheumatology and Immunology outpatient clinics. Exclusion criteria for healthy and patient participants comprised of fracture of hand and finger bones in the five years before entry in the study and distinct destruction of the finger joints. Disease activity was assessed using the erythrocyte sedimentation rate (ESR), see [45], C-reactive protein (CRP), tender/swollen joint count 78/76, and Disease-activity-score (DAS)-28, see [46]. Patient reported disease activity was recorded using the visual analog scale (VAS) for global disease activity and HAQ. The assessment of clinical hand function included three components. First of all, isometric grip strength was measured in pounds (lbs) using a hand dynamometer (Lafayette Instrument, Lafayette, IN, USA). After a familiarization trial, three measurements of grip strength were performed, starting with the dominant hand and alternating between hands. The highest measured force for each hand was included in the data analysis. Secondly, fine motor skills were assessed using the MPUT [7] which is a validated test procedure for inflammatory diseases [47]. Briefly, subjects are asked to pick up twelve small items and drop them into a box as fast as possible while the time to complete the task is recorded. With each hand two repetitions of the test were completed starting with the dominant hand. The fastest trial for each hand was included in the analysis. Thirdly, subjective hand function was measured using the MHQ, a patient reported outcome measure that scores hand function of the left and right hand [48,49] and has previously been used successfully in RA [5].

#### 2.1.2. Optoelectronic Measurement System

**Figure 1:**
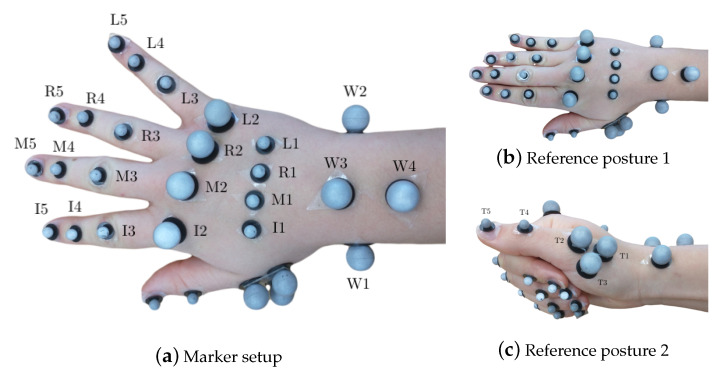
Marker setup showing all 29 markers on the hand in (**a**) with the hand in open position and displaying the marker labels and in (**b**) with the hand in the first flat reference posture and in (**c**) with the hand in the second reference posture displaying thumb marker labeling.

Hand segment kinematics were recorded with a 29 retroreflective spherical marker layout, described in [16], with diameters of 8 mm and 14 mm at a frame-rate of 100 Hz. Reduced marker layouts, such as [50,51] have been presented in literature. However, the analysis required of their outputs requires higher efforts to compute joint angles, which far outweighs the effort to use a 29 marker layout. Furthermore, since the measurements were performed for a high number of subjects for a pilot study, it was desirable to begin with acquiring measurements with highest possible accuracy, before progressing to reduced layouts. The markers were tracked by synchronized and calibrated high-resolution and high-speed infrared cameras (eight Oqus7+ cameras and one Oqus5+ camera, Qualisys AB, Sweden). They were placed on the hand dorsum using double-sided hypoallergenic adhesive tape, as shown in Figure 1a. As a variation from [16], the thumb metacarpal (MC) was tracked using a T-cluster with 3 markers [52], and a marker each on the interphalangeal (IP) joint and the thumb tip was used. The thumb markers were labeled as T1 (MC base), T2 (MC head), T4 (IP joint) and T5 (thumb tip), see Figure 1c. The third marker in the T-cluster was named T3. Furthermore, since RA deformities are primarily observed in the finger MC heads, along with their small relative motion in precision grasping, 14 mm markers were used to allow for better placement and tracking, as compared to the setup in [16].

#### 2.1.3. Doppler Radar

**Figure 2:**
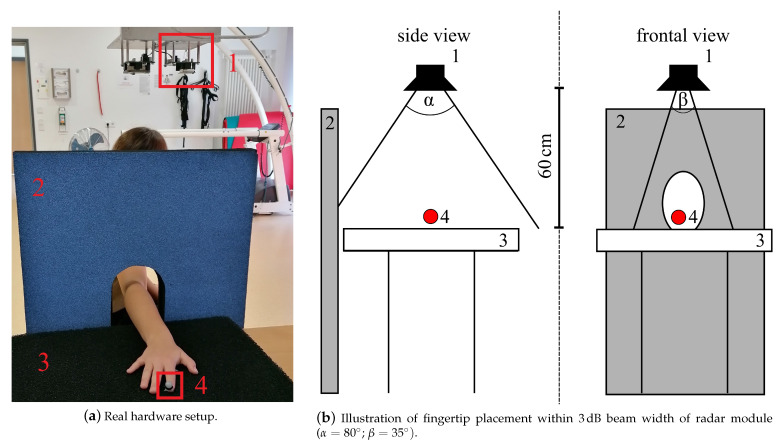
Radar measurement setup: 1—CW radar, 2—absorber wall, 3—absorber mat on table, 4—optical marker placed on fingertip to collect reference data.

The radar measurement setup used in this study to measure a simple finger movement described in Section 2.2.3 consisted of a 24 GHz CW radar, which was originally designed as a vital signal radar, one absorber mat as well as one absorber wall to reduce disturbing reflections of the incident radar wave, see Figure 2a. An optical marker was placed on the respective fingertip (see Figure 1a I5 and L5) to generate reference data with the OMS, see Table 1 set E. Both systems were synchronized using a common trigger signal. The distance between the absorber mat and the monostatic radar module (RSM2650, B+B Thermo-Technik GmbH) was approximately 60 cm. The respective finger was positioned within the 3 dB beam width, which describes the angle between the points of half power of the radar main lobe, and moved along the line of sight of the radar. The horizontal and vertical beam widths were $80{}^{{}^{\circ}}$ and $35{}^{{}^{\circ}}$ respectively. The placement of the optical fingertip marker within the 3dB beam width can be seen in Figure 2b. As a first step of the analog signal processing, the received radar signal was down-converted using an In-phase and Quadrature (IQ) demodulator. The analog signal processing also included a low-pass-filter with a cutoff frequency $f_{\mathrm{LP}}=$ 50 kHz to get the baseband signal and to filter out unwanted signal components, which arise due to the IQ demodulation, as well as a high-pass-filter with a cutoff frequency $f_{\mathrm{HP}}=$ 160 mHz that eliminated the DC offset and signal components from stationary targets. After filtering, the radar data was sampled at 13 kHz with an analog-to-digital converter. This was followed by the digital signal processing in Section 2.3.3, which e.g., included further filtering processes, to extract the fingertip speed of the recorded signals.

#### 2.1.4. Electromyography Measurement System

**Figure 3:**
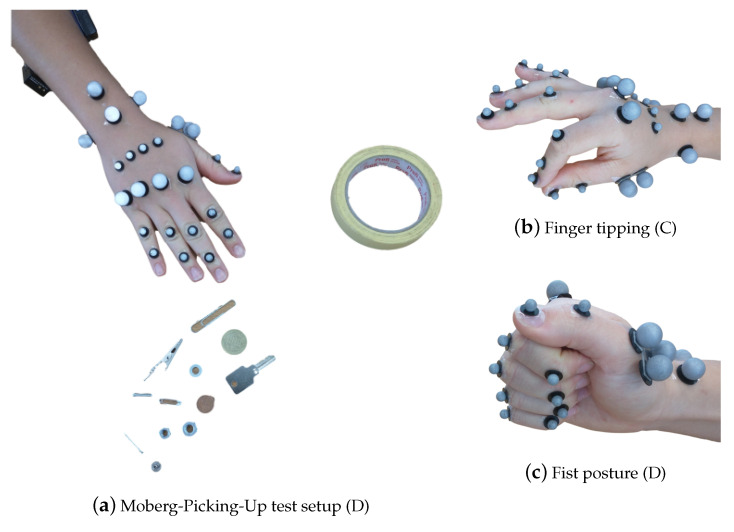
The hand postures for the different recordings, listed in Table 1, with the respective marker set-up. In (**a**), the 25 marker set, along with the surface EMG sensors, as described in Section 2.1.2, for the MPUT. The participants are instructed to lift and place 12 objects in the nearby container. In (**b**), finger tipping motion is shown between the thumb and the index finger with a 29 marker set. In (**c**), the fist posture showing the full flexion capacity of the hand is demonstrated.

Further insight into the RA effects on the hand musculotendon network was obtained by the measurement of muscle activity with two surface EMG sensors (Noraxon Desktop DTS dual electrodes) while patients performed the different activities in sets C and D in Table 1. The electrodes were placed on the flexor carpi radialis and extensor carpi radialis muscles on the dorsal and ventral sides of the forearm, respectively [53], as seen in Figure 3a. The EMG system measures muscle activation in voltage units, specifically, mV. When the measurements will be used for analysis later, the activation will be normalized with respect to the maximum voluntary contraction, see [54], performed during the grip strength test.

### 2.2. Data Collection

#### 2.2.1. Clinical Data

Data collection of clinical information for all patient participants was conducted in combination with a routine visit to the outpatient clinics of the Internal Medicine 3, University Hospital Erlangen. Healthy control subjects were scheduled for a complete test session. Test sessions started with collecting anamnestic data and performing clinical hand function tests described above, in Section 2.1.1.

#### 2.2.2. OMS Data

Participants’ hands were equipped with the reflecting markers and OMS data collection started with the measurement of the reference postures (RPs), set B in Table 1, followed by other physiological tests. The RPs are two neutral repeatable positions for the hand, which define the relative zero angles between two successive segments, see [16]. These positions were, therefore, used to compare the joint angles obtained in two separate measurements, either with the same patient or different patients. The first RP (RP1) defined the relative position of the fingers, excluding the thumb, with respect to the wrist. Here, the forearm and hand lay on a flat surface, keeping the fingers close together and the forearm markers W3 and W4 were aligned with the middle finger metacarpal markers M1 and M2, see Figure 1b. RP2 was used to define the thumb neutral posture. The thumb distal phalanx was rested on the lateral side of the index finger medial phalanx, with the fingers flexed and in a relaxed posture, as shown in Figure 1c, such that the thumb markers T1, T2, T4 and T5 were aligned in a straight line with the wrist marker W1, when viewed from the top. For the measurements in sets C and D, the participants were additionally equipped with the EMG measurement setup. In the first test in set C, named joint relation, the participants were asked to start with an open handed posture, and flex the finger IP joints three times. This was done to estimate relations between the finger IP joints for the different fingers, similar to the common relation in the literature [55], i.e., $\theta_{\mathrm{DIP}}=\frac{2}{3}\theta_{\mathrm{PIP}}$. This served a primary purpose as a comparative measure among the different healthy and RA individuals, along with a secondary utility to estimate the $\theta_{\mathrm{DIP}}$ angles for the different fingers in case the I5, M5, R5, and L5 markers are removed.

Then, finger motion for touching the thumb with each of the fingers, as shown in Figure 3b, named here as finger tipping was recorded. The sequence was performed three times. After that, an activity to measure the finger synergistic kinematic coordination, see [56,57], by performing grasping of objects of two shapes, namely, spherical and cylindrical was performed. The participants were asked to passively, i.e., with application of minimal force, grasp and lift three objects with different dimensions for a single shape. In doing so, movements were recorded for performing the Power Sphere, the Medium Wrap and Prismatic 4 Finger grasps, from the grasp taxonomy, see [58]. Following on to set D, firstly the participants were asked to make a fist, to evaluate the maximum kinematic flexion capacity of the fingers starting from an open posture. After that, the grip strength test and the MPUT were repeated with the markers and EMG sensors. For this set, the markers I5, M5, R5, and L5 are removed. This was done to avoid reflections between the markers themselves during the fist and grip strength tests, and between the markers and the objects, while grasping with precision in the MPUT. In the set E, we used two single markers I5 and T5 to record the finger tapping motion, which is described in the following subsection.

#### 2.2.3. Doppler Radar

**Figure 4:**
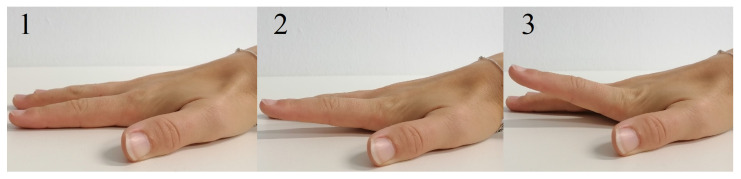
Hyper-extension of the index finger to perform tapping movement (sequence 1-2-3-2-1).

To evaluate the application of radar for the assessment of hand movement, participants were asked to perform a simple finger motion consisting of a periodic hyper-extension of the index and little finger, named finger tapping, while the hand lay flat on the table, see Figure 4. In one measurement scenario all participants were asked to perform the finger tapping as fast as possible (frequency tapping), in another one they were instructed to focus on the maximum deflection of the finger performing the movement in their own speed (amplitude tapping). The parameters of interest were the maximum occurring upward and downward angular velocity of the finger in case of the frequency tapping movement as well as the maximum occurring deflection angle while performing the amplitude tapping movement. To measure the frequency tapping sequences, both sensor systems, radar and OMS, were used. In the following though, only the frequency tapping of the index finger was evaluated as an example. Furthermore, the amplitude tapping was also recorded by both sensor systems, but since the movement was not a continuous motion, important signal components were filtered out by the analog high-pass-filter. Therefore, the evaluation of those measurements was only done using the OMS data.

### 2.3. Data Processing

#### 2.3.1. Clinical Data

Clinical data from the routine visit of the same day was included in the data analysis. In this analysis, anthropometric data and disease activity will be reported, as well as the results of the clinical hand function assessments.

#### 2.3.2. Marker Data Processing

The trajectories of the captured markers was visualized in 3D space by the optical motion capture system from the Qualisys Track Manager (QTM) (https://www.qualisys.com/software/qualisys-track-manager/ (accessed on 5 February 2021)). The marker tracking in QTM was performed using the Automatic Identification of Markers (AIM) module, to identify different labeled positions. QTM allows for calculation of Euler Angles between two rigid bodies, where every body is defined by three markers. This was not feasible for our measurements, where every segment was defined by a single marker, except for the thumb metacarpal. The marker coordinates were processed to calculate the joint angles, in particular the flexion-extension (F-E), adduction-abduction (A-A) and pronation-supination (P-S) angles, between different segments in the following way. A body-fixed coordinate system was setup for every segment using the description provided in [16], with the underlying assumption that the rotation axes are perpendicular to the segments’ anatomical planes. In accordance with the recommendations by the International Society of Biomechanics (ISB), see [59], the X-, Y- and Z-axes were positive along the palmar, proximal and radial directions, respectively. The relative rotation matrix between two subsequent segments was then calculated for every position. The rotation matrix was subsequently used to calculate the Tait-Bryan or Cardan angles with the order ZXY, or F-E, A-A, followed by P-S. The procedure to calculate joint angles was performed for all measurements. For the tests in set D in Table 1, the PIP joint angles were determined with the IP joint angle relations evaluated in set B. Additionally, to assist the radar processing, the index and little finger metacarpal hyper-extension motions were recorded using the vertical position of the I5 and L5 markers, respectively. The hyper-extension motion is used to calculate the maximum possible angular velocity, as well as the hyper-extension angle for the two fingers. To compare these quantities across subjects with different hand dimensions, we calculated the finger lengths, i.e., for index finger $d_{I2\_I5}=∥I2-I5∥$ and for little finger $d_{L2\_L5}=∥L2-L5∥$, from the RP1 posture.

#### 2.3.3. Radar Signal Processing

The aim of the digital signal processing chain was to extract the fingertip speed from the received radar signal. This was challenging as the radar beam illuminated the entire hand and its surroundings and thus, the complex radar signal resulted from the superimposed reflections of all moving finger segments. The first step of the digital signal processing was the calibration of the IQ imbalance [60]. Afterwards, the DC offset caused by the analog-to-digital converters, was removed and the received signal down-sampled to 1625 Hz, which means that the sampling rate is reduced in order to decrease the size of the data being processed. In addition, the signal was filtered by a zero-phase low-pass-filter with cutoff frequency 320 Hz, eliminating all signal components corresponding to a speed higher than 2 m/s. Afterwards, a short-time Fourier transform (STFT) with Gaussian window, called Gabor transform [61], was applied to the signal. The size of the Gaussian window was set to 512, which corresponds to a time frame of 0.32 s, the standard deviation was 16 and the overlap was chosen to 256 samples. Each window was zero padded by a factor of two. The STFTs of a 5 s cutout of two example measurements can be seen in Section 3. The noise floor of the measurements was −50 dB. Determining the speed of the fingertip became more complex due to window effects, which widen the frequency peaks of the Fourier transform, as well as higher frequency components that were caused by the nonlinear finger movement within one window of the STFT [21]. Thus, in order to detect the fingertip speed from the radar data, it was not possible to simply extract the maximum occurring Doppler frequency and speed respectively. Instead, an amplitude value, which needed to be subtracted from the maximum amplitude per time slot defining a suitable threshold, had to be determined. Then, the maximum speed per time slot, which occurs with an amplitude greater than the defined threshold, could be extracted from the STFT. After that, the extracted curve was smoothed by a low-pass-filter. The selection of the amplitude value was challenging though, as it seemed to be dependent on the shape on the finger of the respective subject as well as on the form of movement. By looking at different measurements and the corresponding STFTs and a simulation of the tapping movement using multiple point scatterers to model the finger, it was possible to empirically adjust the needed amplitude value. The choice of the amplitude value depended on the amplitude ratio between low and high frequencies or speeds. Measurements, in which the amplitude difference between low and high speeds was small, required a low amplitude value. Other measurements, in which higher speeds occured with much smaller amplitude than low speeds, needed a comparatively high amplitude value. To evaluate this behavior, the amount of frequencies and corresponding speeds, which appeared with an amplitude greater or equal to the average amplitude, was set in relation to the number of frequencies appearing with at least 5 dB above noise level. This ratio is called frequency ratio. For all measurements, the amplitude value was set between 7 dB and 25 dB, where frequency ratios above 0.64 lead to an amplitude value of 7 dB and frequency ratios below 0.2 lead to an amplitude value of 25 dB [62]. As an example, Figure 7a in the results section shows all corresponding speeds above noise threshold normalized to the maximum occurring amplitude of −11 dB for one frequency tapping measurement. The minimum and maximum speeds with an amplitude higher than −34 dB (5 dB above normalized noise level) are −0.8m/s and 0.7m/s. Whereas the minimum and maximum speeds with an amplitude higher than the average amplitude of −13 dB (normalized) are −0.42m/s and 0.42m/s. With the given resolution of the Fourier transform of 0.0195 m/s, this results in a frequency ratio (or speed ratio) of 43/77≈0.56 and an amplitude value of 10 dB.

#### 2.3.4. Statistical Analysis

This paper does not present a complete analysis of all outcome measures, as the primary goal is to introduce the scope of measurement techniques compared to and also integrated with conventional clinical evaluations. Data are presented as means, minimum, maximum and standard deviations for the overall study sample and also stratified by study group. The descriptive statistics assume each hand as independent therefore the standard deviations should be regarded accordingly. For a formal comparison of hand function, grip strength and kinematics, we used linear mixed-effects regression models to accommodate the within person clustering of measurements from two hands and the unbalanced data structure, since a valid measurement could not be obtained from both hands in some participants. The models included the respective clinical or kinematics measurement as the dependent variable, study group as the fixed effect where the control group was the reference level and the individual as the random effect. The regression coefficients for the study group term in these models were considered as the effect of RA representing an unadjusted between group difference that accounts for within-person correlation. These were presented with their respective 95% confidence intervals and *p*-values estimated using the Satterthwaite approximation for the model degrees of freedom. The level of significance was set to *p* ≤ 0.05 and was not corrected for multiple testing.

## 3. Results

### 3.1. Clinical Results and OMS Measures Outcomes

Forty-seven individuals participated in this study with a mean age of 56.3 ± 14.2 years (29 females and 18 males). This included 23 healthy control subjects with ages 50.2 ± 16.1 years (12 females and 11 males) and 24 patients with RA with ages 62.3 ± 9.1 years (17 females and 7 males). Mean patient reported disease activity (VAS global disease activity) was 29.4 (standard deviation 25.8). The choice to undertake measurements for either one or both hands provided us with measurement data for $N_{A}=64$ total hands with $N_{C}=35$ healthy controls and $N_{R}=29$ RA patients hands. MPUT times and grip strength results for clinical and OMS setup are summarized in Table 2.

**Figure 5:**
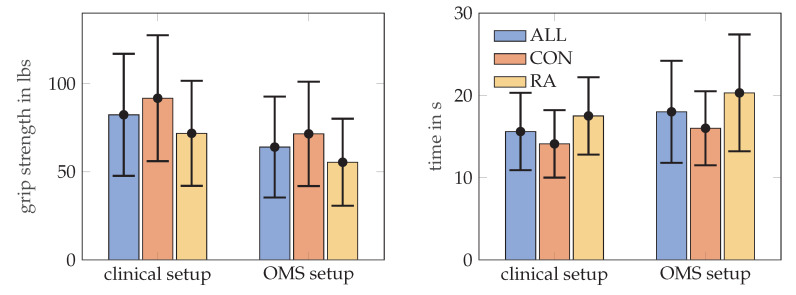
The mean and standard deviation values for the grip strength in lbs, on the left MPUT times, on the right, for all subjects, RA and control (CON) groups. Grip strength was assessed according to the clinical set up and repeated in a sitting position with markers placed on the hand (OMS setup).

RA patients needed longer to complete the MPUT (17.5 ± 4.7 s) compared to control subjects (14.1 ± 4.1 s). Mean MPUT times with markers during OMS data collection was 20.3 ± 7.1 s for RA patients and 16.0 ± 4.5 s for the control group. This increase in MPUT times is similar for control and RA participants and also for men and women. Figure 5, illustrates the MPUT times for the two groups. The mean, standard deviation, maximum and minimum values values for MPUT and grip strength test are provided in Table 2. Group coefficient from a mixed model indicating between group difference after accounting for within person clustering is presented in Table 3.

In Table 4, the mean, standard deviation, maximum and minimum values for angular velocities, number of cycles and maximum hyper-extension angles are provided for the tapping motion across the two groups. The hyper-extension angles can be computed, e.g., for the index finger, as the inverse cosine of the ratio between the maximal vertical distance covered by the marker I5 on the finger tip and the finger length $d_{I2\_I5}$, defined in Section 2.3.2. Table 3 provides the unadjusted absolute RA-Effect (absolute difference RA vs. Control) differences with 95% confidence intervals (CI) and *p*-values from mixed-models analysis.

**Figure 6:**
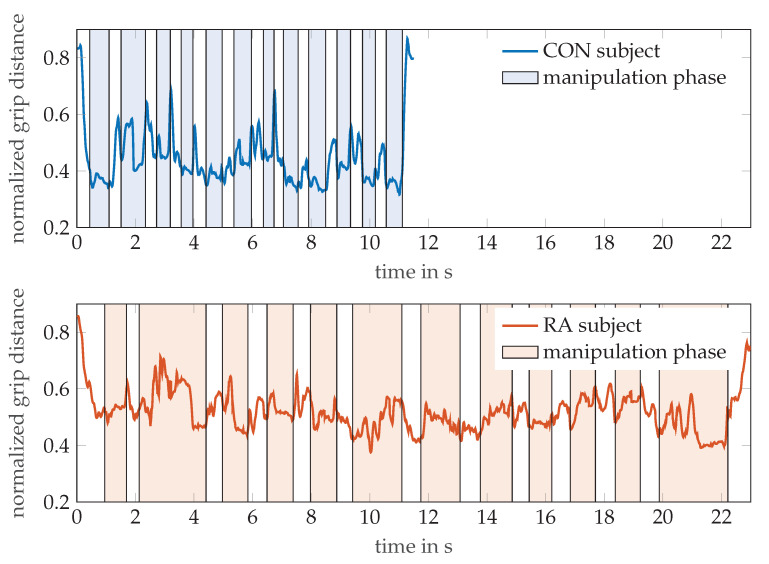
The normalized grip distances between thumb tip (T5) and index finger DIP (I4) markers are shown for a participant each from the control and RA groups, while performing the MPUT. The shaded and non-shaded areas in each plot correspond to the manipulation and prehension motions, respectively.

Figure 6 illustrates the additional information that can be gained from the MPUT by OMS tracking of the hand motion. The graph exemplarily shows the evolution of the distance between markers T5 and I4 (normalized to finger length) during pick and drop actions of the hand while the respective participants perform the MPUT. For a healthy control individual (age 32, male) and an RA patient (age 82, female), the distance between these markers was normalized with respect to their finger lengths, as done for the calculation of the tapping motion. This was plotted over time overlaid by the time periods of the manipulation and prehension motions observed from the video. The shaded and non-shaded regions in the plots refer to the manipulation and reaching motions, respectively. While a traditional MPUT analysis would involve comparing only the fastest times, a sensor based recording with hand kinematic data allows more observations. For instance the mean ± standard deviations values for the distance between I4 and T5 are 0.45 ± 0.11 and 0.52 ± 0.07 for the control individual and an RA patient, respectively. This suggests that the control individual was able to maintain the two fingers closer (lower mean), and was able to perform prehension with a higher degree of mobility (higher standard deviation), when compared with the RA patient.

### 3.2. Radar Results

**Figure 7:**
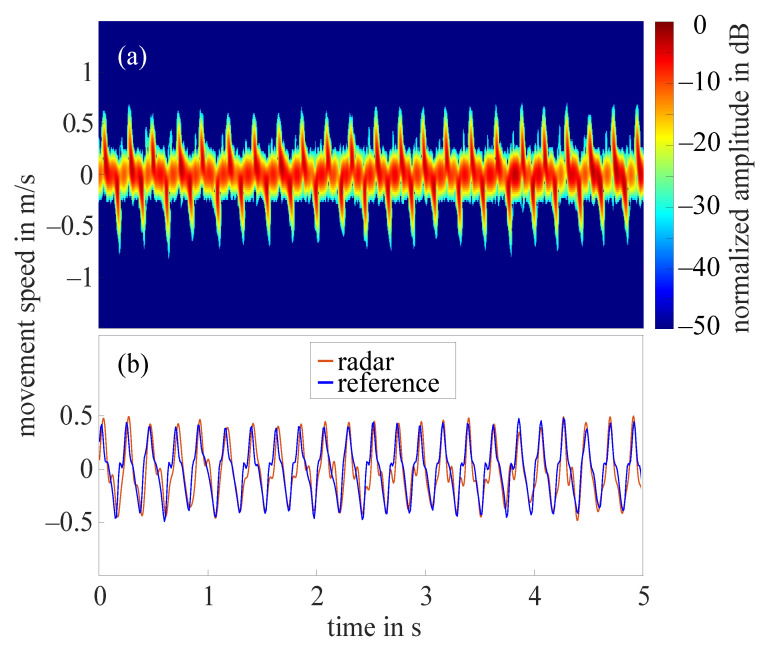
(**a**) Short-time Fourier transform of an example measurement with amplitude value 10 dB; (**b**) extracted fingertip speed of radar measurement shown in (**a**) and reference data collected by OMS.

**Figure 8:**
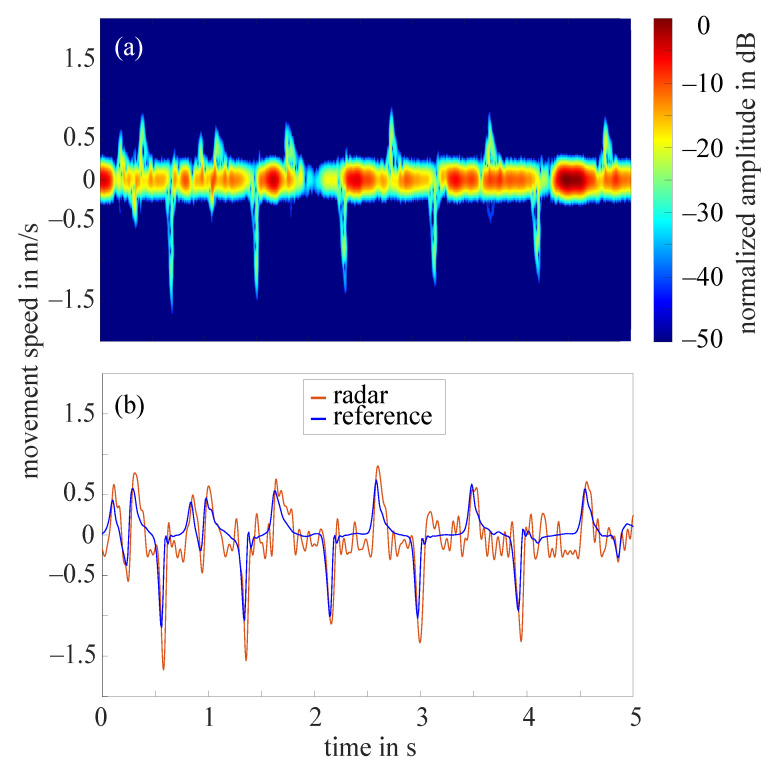
(**a**) Short-time Fourier transform of an example measurement with strong amplitude fluctuations and a selected amplitude value 20 dB; (**b**) extracted fingertip speed of radar measurement shown in (**a**) and reference data collected by OMS.

The frequency tapping of the index finger was measured by the radar for 62 hands in total as in two cases the radar did not measure properly. Figure 7a shows the STFT of a 5 s cutout of an example measurement that required the amplitude value to be set to 10 dB. The amplitudes were normalized to the maximum occurring amplitude (−11 dB), all amplitudes below noise threshold were excluded. The extracted fingertip speed compared to the reference data collected by the OMS can be seen in Figure 7b. The comparison of both curves illustrates that the radar fingertip speed is almost congruent with the reference data. However, the radar signal processing presented here reaches its limits as soon as larger amplitude fluctuations occur over the measurement time. Figure 8a shows the STFT (normalized to the maximum occurring amplitude, 5 s cutout) of such a measurement. The automatically selected amplitude value would be 20 dB in this case. The extracted radar fingertip speed compared to the OMS reference data can be seen in Figure 8b. It should be noted that due to the analog high-pass-filter, speeds below 1 mm/s were filtered out which causes the fluctuations of the radar fingertip speed in this area. Furthermore, the two curves show that the selected amplitude value did not yield an optimal result for the entire measurement, as the maximum deviation between radar data and OMS is approximately 0.5 m/s. These amplitude fluctuations are most likely caused by an unfavorable finger position under the radar module. Future measurement campaigns should include an automatic detection of such faulty measurements, to ensure that measurements can be repeated in this case. Since this was not given in the current setup, 14 measurements had to be discarded due to those amplitude fluctuations.

To evaluate the quality of the remaining 48 radar measurements, a relative error concerning the maximum occurring upward fingertip speed in comparison to the maximum occurring upward fingertip speed detected by the OMS was calculated. The mean value of the relative error for all 48 measurements was 11%.

## 4. Discussion

The objectives of this study were to design an experimental framework that allows to comprehensively assess hand movement in patients with rheumatoid arthritis. The study integrated clinical and sensor data with the goal to evaluate the potential of radar to serve as a markerless sensor for the acquisition of hand movement. While the application of optical marker tracking for the quantification of hand movement will already broaden the understanding of the role of hand function for the identification of disease activity, the use of modern, markerless motion capturing techniques for the assessment of hand kinematics broadens the research scope with an extensive list of measurable attributes, compared to the traditional methods. Furthermore, early assessment of dynamic hand function is favorable when compared to static measurements such as thermal imaging, see [14], or expensive medical technologies such as magnetic resonance imaging.

Recording clinical data and sensor data on the same testing day is essential for the interpretation of sensor data in relation to disease activity. The initially anticipated challenge to find relevant hand movements that are detecTable across the here implemented technologies proved true. The assessed movement needs to suit the deployed technology and at the same time be meaningful in the context of the disease of interest. Assessing hand movement in rheumatoid arthritis patients creates the additional difficulty that hand geometry can be altered.

In this study, the rather complex MPUT was implemented as the common testing scenario between clinical and OMS assessments. As illustrated in Figure 6, compared to the clinical assessment that only includes the time to completion of the task, OMS data would allow to quantify the movement that is needed to complete the picking task and would lead to a movement specific resolution of the time needed. This will allow to identify specific characteristics of a picking task that discriminate patients from healthy individuals. However, the marker set up had to be adjusted by removing fingertip markers as MPUT items frequently stuck to the glue or markers impaired picking up the objects. Marker movement over the joints as a result of skin motion did not prove to be a major challenge in this set up, most likely due to the slow and controlled movements.

For radar data acquisition the movement needed to be rather simple to account for the current scope of the hardware, and also OMS data collection required the recording of very simple reference postures and extremes in the hand range of motion. Slow and rather static postures may however not allow characterization of hand movement to a degree of resolution that is necessary to detect movement patterns in RA patients. For that, intuitive, daily activities may be more relevant.

The post processing of OMS and radar data in the context of hand movements is challenging due to the complex nature of hand motion. The here applied marker set up introduced by [16] allows the extraction of hand movement characteristics and also proved applicable in our patient cohort. Patients performed generally worse in grip strength and MPUT tests when compared to healthy individuals as expected and previously shown [8]. This impaired functionality is also reflected in the here presented kinematic data. While the kinetic analysis of hand movement will provide parameters that allow a more detailed characterization of hand function in relation to disease status, this study showed, that the time to complete the MPUT protocol is slower in both, patients and healthy controls, when the hands are equipped with the reflecting markers compared to the pure clinical testing. This was observed for women and men equally and emphasizes the fact, that the use of markers artificially alters hand movement and supports the need to develop markerless motion capture systems for unconfined acquisition of hand motion.

While the repetition of the MPUT and grip strength tests with the OMS system is essential to assess the effects of the markers on hand motion and also to visualize, where in the clinical tests healthy controls differ from patient participants, in future analyses of the data set, more simple hand movements, for example grasping spherical or cylindrical objects, will allow a detailed kinematic description of hand movement in RA patients in relation to disease status.

In addition to the MPUT, the angular velocity was evaluated from the measurement of the periodic hyper-extension of the index finger with both the radar prototype and the OMS. RA patients show a reduced mean angular velocity in the MCP joint with an increased standard deviation for the upwards movement compared to the control group which is especially prominent for the upward movement. While this difference is not significant in the statistical results, this needs further investigation in a larger sample size. Especially the difference in the variation between the upwards and downward movement is interesting here, while the latter is assisted by gravity and thus does not require conscious effort, the upward movement may be relevant for distinguishing between healthy and diseased individuals.

To extract the fingertip speed of a moving finger from the radar signal, the signal processing chain presented in this paper was applied. The extraction was challenging as the radar signal measured by the monostatic CW radar module did not allow any spatial resolution and higher frequency components emerged due to the application of a STFT with nonlinear movements within a window. Therefore, a methodology for applying a suitable threshold was introduced in Section 2.3.3. However, the methodology presented could not be applied to measurements, which underlay strong fluctuations in amplitude over the measurement duration. As 9 out of 14 discarded data sets were finger tapping measurements of RA patients, it should be investigated whether this behavior has been caused by geometric differences of RA hands. The detection of the maximum upward fingertip speed using the CW radar showed an error of 11%, which implicates that the presented CW radar hardware setup and signal processing chain are suitable to some extent. Anyhow, to reduce the relative error, the current signal processing chain needs to be further improved. Using the parameter frequency ratio to determine the amplitude value could be improved by introducing a sliding amplitude value that is variable over the measurement time. In case the form of the finger movement changes within one specific measurement (e.g., in between upward and downward finger movement), an adjustment of the amplitude value for every second or less is likely to benefit the signal processing. In order to completely avoid the problem of determining an amplitude value and threshold, the use of more complex radar systems with high angular resolution, which allow to resolve different finger parts, has to be investigated in this context. In addition, to measure more complex hand movements, radar systems with high spatial resolution (angular and range) are essential and should therefore be part of future research. In summary, radar is a promising contact- and markerless option to be used for the objective assessment of hand function as well as for controlling therapeutic success.

Overall, more women then men participated in the study. While the sex distribution in the control group was even with 12 females and 11 males, more female RA patients were recruited compared to males (17 females vs. 7 males). Even though this reflects the sex distribution of RA patients in the general population, given the fact that the effect of disease on hand function varies between sexes, a more targeted subject recruitment to achieve equal distributions of ages and sexes between control and patient participant should be aimed for. Furthermore, the integration of clinical and sensor based data will require larger sample sizes that account for the greater variation in movement patterns observed in the patient population.

## 5. Conclusions

The paper emphasizes the need for adapting newer technologies to assist in the characterization of hand movement in patients suffering from RA. To achieve this, the integration of existing clinical methodologies together with state of the art technologies and experimental methodology is essential. Optical tracking using OMS has been shown capable of capturing a variety of hand movements observed in activities of daily living. However, differences in the results noticed due to the use of surface markers attached to the subjects’ skin surfaces can be circumvented through the markerless radar technology. The preliminary examinations carried out show a promising potential for the development of sensory recordings of hand movements using radar technologies.The comparison of finger motion data collected by the marker-based OMS to the data recorded by radar implies that even a simple monostatic CW radar can be used to measure simple hand motions. Furthermore, due to the difference in MPUT times with and without markers, a non-contact and marker-free measurement method is clearly preferable for these measurements.

## Figures and Tables

**Table 1:** Overview of the complete testing procedure for one patient/hand.

	Test	Repetitions	Markers	EMG	Radar
A	grip strength	3	-	-	-
	Moberg-Picking-Up Test	3	-	-	-
B	reference posture 1	1	29	-	-
	reference posture 2	1	29	-	-
C	joint relation	1	29	2	-
	finger tipping	1	29	2	-
	grasping: spheres	1	29	2	-
	grasping: cylinders	1	29	2	-
D	fist	1	25	2	-
	grip strength	2	25	2	-
	Moberg-Picking-Up Test	2	25	2	-
E	tapping index finger: frequency	1	1	-	1
	tapping index finger: amplitude	1	1	-	1
	tapping little finger: frequency	1	1	-	1
	tapping little finger: amplitude	1	1	-	1

**Table 2:** The table provides the mean (sd, or standard deviation), minimum, and maximum values for subjects’ grip strength and times for MPUT in the clinical setting and with the OMS setup.

		ALL ($N_{A}=64$)			CON ($N_{C}=35$)			RA ($N_{R}=29$)		
		min	mean (sd)	max	min	mean (sd)	max	min	mean (sd)	max
grip strength in lbs	clinical	32	82.3 (34.6)	178	44	91.7 (35.7)	178	32	71.8 (29.8)	134
	OMS	19	64.0 (28.6)	140	19	71.5 (29.6)	140	20	55.4 (24.7)	102
MPUT times in s	clinical	9.2	15.6 (4.7)	31.4	9.2	14.1 (4.1)	31.4	12.2	17.5 (4.7)	30.1
	OMS	11.1	18.0 (6.2)	41.0	11.1	16.0 (4.5)	31.9	12.2	20.3 (7.1)	41.0

**Table 3:** The table provides the unadjusted absolute RA-Effect (absolute difference RA vs. Control) differences with 95% confidence intervals (CI) and *p*-values from mixed-effects regression models for the outcomes reported in Table 2 and Table 4. *p*-Values < 0.05 indicate a significant difference in the respective outcomes between RA and Control participants. The regression coefficients for the study group term in these models were considered as the RA-effect representing an unadjusted between group difference that accounts for within-person correlation.

		Outcome	RA-Effect, 95% CI		*p*-Value
clinical		MPUT time in s	3.87	(1.36 to 6.39)	0.004
		grip strength in lbs	−23.61	(−42.34 to −4.88)	0.017
OMS		MPUT time in s	5.07	(1.56 to 8.57)	0.007
		grip strength in lbs	−19.62	(−35.01 to −4.23)	0.016
	index finger	ang. vel. up in deg/s	−14.37	(−82.05 to 53.31)	0.679
		ang. vel. down in deg/s	−2.95	(−85.06 to 79.16)	0.944
		num. cycles	−4.58	(−9.96 to 0.81)	0.103
		hyper-ext. in deg	−4.46	(−10.12 to 1.21)	0.131
	little finger	ang. vel. up in deg/s	−29.42	(−86.50 to 27.67)	0.318
		ang. vel. down in deg/s	−9.41	(−72.99 to 54.17)	0.773
		num. cycles	−7.79	(−14.96 to −0.62)	0.039
		hyper-ext. in deg	−3.53	(−9.50 to 2.44)	0.253

**Table 4:** The table provides the mean value, standard deviation, minimum, and maximum values for different quantities for subjects’ tapping motion. These include the angular velocity (ang. vel.) in the vertically upwards and downwards directions, and number of lifting cycles (num. cycles) in the frequency tapping exercise. The hyper-extension (hyper-ext.) angle is calculated for the amplitude tapping exercise.

		ALL ($N_{A}=64$)			CON ($N_{C}=35$)			RA ($N_{R}=29$)		
		min	mean (sd)	max	min	mean (sd)	max	min	mean (sd)	max
index finger	ang. vel. up in deg/s	118	381 (113)	729	174	381 (113)	729	118	371 (124)	598
	ang. vel. down in deg/s	187	438 (142)	760	228	436 (125)	713	187	441 (160)	760
	num. cycles	15	46.0 (10.2)	82	33	48.0 (9.5)	82	15	43.5 (10.4)	58
	hyper-ext. in deg	13.4	38.1 (10.4)	68.4	20.2	40.0 (10.8)	68.4	13.4	35.6 (9.3)	51.3
little finger	ang. vel. up in deg/s	87.1	237 (100)	562	87.1	248 (101)	562	90.5	225 (97)	461
	ang. vel. down in deg/s	127	317 (116)	675	127	319 (113)	675	127	315 (120)	579
	num. cycles	5	33.5 (13.1)	63	7	37.1 (10.0)	63	5	29.0 (13.6)	57
	hyper-ext. in deg	8.4	26.4 (10.8)	57.5	8.4	27.9 (10.8)	53.9	10.6	24.6 (10.4)	57.5

## Data Availability

Data is available upon request.
